# Supplementary material for: A new shortcut for competitive sports development? The purpose of and strategy for developing and introducing sepaktakraw in Taiwan
Source: PLoS One. 2025 Jan 13;20(1):e0317451. doi: 10.1371/journal.pone.0317451 (PMC11729932; doi:10.1371/journal.pone.0317451)
Supplement: Attachment — Submitted filename: Response to Reviewers.pdf [file pone.0317451.s001.pdf]

Response to Reviewers

Reviewer #1: This manuscript investigated the introduction and development of a new sport, sepaktakraw, in Taiwan. The SPLISS model was appropriately used. This study provided detailed insights as several key members of the sport were interviewed. This study also identified a major reason for the local government to support this sport, ie, winning medals in international competitions. This aspect is different from many western countries. Several minor points need to be addressed.

#1 Reviewer's comments to the author	Response and amendments to reviewer' comments
1. The sentence 'after the new president took office in 2018' has been used multiple times throughout the manuscript. Please modify or simply.	Thank you for your suggestions. We have made changes throughout the text regarding the sentence 'after the new president took office in 2018'. Please refer to lines 326, 414, 439, and 473.
2. Line 124: Chinese Taipei 'Sepak Takraw' Federation or Chinese Taipei 'SepakTakraw' Federation? Also in line 183-184.	We are grateful for the your suggestion. We have revisited the CTSTF website to confirm that it is the Chinese Taipei Sepaktakraw Federation. We have reviewed the entire text for corrections where the Chinese Taipei Sepaktakraw Federation appears. Please refer to lines 164-165, 712, 734–735, 739, 744–745, 747–748, 750, 754–755, 771–772, and 778.
3. Line 133: Sports Administration, Ministry of Education.	Thank you for your suggestion. We have added the missing comma. Please refer to line 187.
4. Line 218: In 2010, sepaktakraw received widespread attention. Please be more specific on 'widespread attention', such as the number of participating countries or athletes.	Your suggestions were greatly appreciated. Due to the lack of clarity in the previous statement, which caused misunderstanding among the reviewers, we would like to clarify the following: According to Interviewees B and S, sepaktakraw has received much attention from the Taiwanese community. This is due to the 2009 World Games being held

	in Taiwan. The revised text reads as follows: The former president of the CTSTF and its administrator claimed that "since the 2009 World Games, the sport of sepaktakraw has received considerable attention in Taiwan, which has led to an increase in government subsidies compared with the CTSTF's self-financing" [Interviewee B; S]. See lines 320–322.
5. Line 230-231: 2018 World Sepaktakraw Championships of 'division' 1…?	Thank you for the observation. The missing 'd' has been added as a result of our oversight. Please refer to line 332.
6. Line 233: …government funding has increased dramatically. Please be more specific on the number of funding.	Thank you to the reviewers for proposing the changes. A specific amount of increased funding has been added there. Please refer to lines 334–335.
7. Line 234-236: The best international competition result was achieved in 2015, but the government budget peaked in 2019 and was decreasing since. Please clarify.	Thank you for your suggestion. In an effort to explain the decrease in funding, we have added a note explaining that tournament hosting and international tournament attendance were suspended due to the COVID-19 epidemic. Please see lines 336-338.
8. Line 268: sending athletes to PE departments and serving as club coaches seem to be unrelated. Please clarify.	Thank you for your suggestion. The text has been adjusted to explain how and why CTSTF increases sport participation at an early stage. Please see lines 377–389.
9. Line 270: …ensure these athletes will become PE teachers…Attending normal universities does not guarantee these athletes will become PE teachers. Please revise.	Thank you for your suggestion. According to the reviewer's suggestion, we have revised the formulation to include three goals: developing a force ready for international competitions, coaching in nearby primary and secondary schools in their spare time, and the possibility of becoming a full-time PE teacher in the future, teaching on school campuses and organizing competition teams. Please see lines 377–389.

10. Line 283-285: please be more specific on which year(s) was 'now' and 'early years'.	Thank you for your suggestion. The sentence has been revised to include the precise year. Please refer to line 400.
11. Line 351: …countries were widely invited to compete in Taiwan. Please be more specific on the number of countries invited.	Thank you for your suggestion. Data have been re-examined and specific countries that participated in the competition have been added. Reference lines 476 to 477.
12. Line 384: …Mr. 'Huang' Chung-Jen…	Thank you for the observation. His surname has been added. Please refer to line 517.
13. Figure 1: the background and numbers in Y-axis can be modified according to the Journal's style.	Thanks to the reviewer for the suggested amendments. We have referenced https://journals.plos.org/plosone/s/figures#loc-preparing-figures-from-image-files to make the correction. Figure 1 shows the standardized font form and background removal. Please refer to the amended figure.

Reviewer #2: Dear Author！

After careful review of this article. Read through, this article explores why Taiwan introduced an emerging sport, rattan ball (sepaktakraw), and how it achieved international sporting success in the short term. The study uses the sport policy model proposed by De Bosscher et al. as a framework for analysis. Using official government and sports federation documents, media reports, and semi-structured interviews with 18 key stakeholders, the study identified three main reasons for the introduction of rattanball: (1) low cost of introduction; (2) medal breakthrough at the Asian Games; and (3) facilitating access to the leadership of international sports organizations. The development strategy of rattan in Taiwan includes seven aspects: obtaining government financial support, institutionalizing organizational decision-making, using universities as a breakthrough point and gradually expanding to the grassroots level, selecting players based on tournament results, utilizing school badminton courts, enhancing coach training, and actively participating in and striving for the hosting of international tournaments. In addition, the study raises four concerns: the contingency and inevitability of the introduction of new competitive sports, the trajectory of development, the strategy of cross-

sport talent selection, and the issue of sustainability. However, the article's argumentation process is too uncritical and there are areas that need to be revised.

#2 Reviewer’s comments to the author	Response and amendments to reviewer' comments
1. Clarity and conciseness: in the abstract, improve scholarship. Abstract required before introduction section also required to write properly. It should be written in the following steps;<br>a) Write the concept as per your paper title (2 lines minimum).<br>b) Objective (2 lines)<br>c) Tools (2 lines)<br>d) Output (3 lines)<br>e) Output with application in real life or in the industry (mandatory)	Thanks to the reviewer for the specific suggestions for amending the abstract. Based on the elements that the reviewer pointed out that should be included in the abstract, as well as parts a and e that were not included in the original abstract, we have added sections a and e to the abstract. Please refer to lines 16-17 and 30–32.
2. Deepening of the theoretical framework: to consider further generalising and expanding the existing theoretical framework, it is recommended that a more in-depth literature review of existing research be conducted to highlight the importance and innovation of this study in the current field of academic research.<br>In this section,<br>a) the background of this research domain is way sufficing and the justification for this research (add some proper implication	Regarding the literature review, we thank the referees for their specific suggestions, and we have made the following amendments:<br><br>1. The literature review and introduction have been revised to include and highlight the research gaps as well as references to relevant areas provided by the reviewer (in response to the referees' comments a, b, c, and g). Please refer to lines 47–53, 76, and 126–143 for more details.<br>2. On the basis of the most recent literature, we have added new paragraphs to the literature review about the strengths and

fig./picture in the background)<br>b) research gap, weakly presented.<br>c) the proposal of this research does not seem to be clearly presented to address the research gap. Please revise the Introduction section accordingly.<br>d) Literature review shall be comprehensive (rather than brief) to discuss the right breadth of knowledge and recent works in the area.<br>e) Better to add more recent work in the contribution table.<br>f) Author should provide the real case study with an appropriate diagram/picture to explain with details.<br>g) Managerial implications would be useful.<br>In conjunction with analysing articles in related fields, you can refer to relevant literature on public participation and public complaints to enhance the theoretical support of the introductory section, highlight the theoretical orientation, focus on the argument from a theoretical perspective, and put forward relevant theoretical support. The following articles can be highlighted for reference:<br>①Di, K., Chen, W., Shi, Q., Cai, Q., & Liu, S. (2024). Analysing the impact of coupled domestic demand dynamics of green and low-carbon consumption in the market based on SEM-ANN. Journal of Retailing and Consumer Services, 79,	specifically why the SPLISS model should be used (in response to reviewer comments d and e). Please refer to lines 92–106.<br>3. In the literature review section, we updated the SPLISS model according to the latest literature and discussed management implications (in response to the reviewers' comments d, e, f, g). Please refer to lines 92–106 and 126–143.

	103856.<br>②Di, K., Chen, W., Zhang, X., Shi, Q., Cai, Q., Li, D., ... & Di, Z. (2023). Regional unevenness and synergy of carbon emission reduction in China's green low-carbon circular economy. Journal of Cleaner Production, 420, 138436.<br>③Di, K., Chen, W., Shi, Q., Cai, Q., & Zhang, B. (2024). Digital empowerment and win-win co-operation for green and low-carbon industrial development: Analysis of regional differences based on GMM-ANN intelligence models. Journal of Cleaner Production, 141332.	
3.	Clarity and Structure: The article is well-structured, with a clear introduction, methodology, results, and discussion sections. However, some parts could benefit from more explicit transitions between sections to improve the overall flow.	Thanks for your suggestions. At the end of the introduction, literature review, and methodology, we have added an introductory sentence about section-to-section conversion. Please refer to lines 61–62, 150, and 213–215.
4.	Literature Review: While the study adopts De Bosscher et al.'s model as the analytical framework, a more detailed explanation of this model and its relevance to sepaktakraw's introduction in Taiwan would strengthen the theoretical foundation.	Thank you for your suggestions. In the literature review, we not only discussed the recent adoption of the SPLISS model by scholars but also linked it to sepaktakraw's introduction in Taiwan. Lines 126–143 are relevant.

5. Data Collection and Analysis: The use of semistructured interviews with 18 key stakeholders is appropriate, but more information on the selection criteria for these stakeholders and the interview process would enhance the credibility of the findings. Additionally, detailing how media reports and official documents were analyzed could provide more transparency.	Thanks for your suggestions. In the data and analysis section of the methodology, we have adjusted the discussion of the rationale for adopting a longitudinal research design, the criteria for specifying the selection of stakeholders, the important characteristics of the two groups of respondents, and information about the interview process, such as the location, the delivery of consent forms, and the validation of the interview data. We also describe how we analysed the relevant textual data in this study in the section on data analysis. Please see lines 166–180, 185–186, 190–201, and 205–215 for more information.
6. Findings and Discussion: The three main reasons for introducing sepaktakraw in Taiwan are clearly stated, but providing more context or examples for each reason would make the findings more compelling. For instance, elaborating on the specific costs saved, or success stories of other countries in similar positions, would add depth.	Thanks for your suggestions. We have re-examined the three main reasons for sepaktakraw's introduction to Taiwan. We have provided relevant cost savings for each reason, and supplemented this with examples from other countries or sports. This contrasts with the reasons for sepaktakraw's introduction to Taiwan. See 224–240, 242–252, and 284–288.
7. Development Strategy: The seven prioritized aspects of the development strategy are comprehensive. However, the article could benefit from discussing potential challenges or limitations associated with each aspect. For instance, the practical difficulties in using school badminton courts or obtaining sustained government financial support could be addressed.	Thank you for your suggestions. At the end of the seven areas of development strategies, we have attempted to discuss potential challenges or constraints to sepaktakraw development in Taiwan. Diversifying funding, expanding professional facilities, and including the sport in representative tournaments and competitions are some

	examples. Refer to lines 340–343, 368–373, 401–404, 424–431, 446–448, and 483–489.
8. Issues of Concern: The four issues of concern raised are critical and thought-provoking. To enhance the discussion, incorporating comparative examples from other countries that have introduced new sports could provide a broader perspective on the contingency and inevitability, developmental trajectory, cross-sports talent selection strategy, and sustainable development issues.	Thanks for your suggestions. The discussion section, originally devoted to Taiwan's situation, has been expanded to include other countries as comparative examples. As a result of the changes in the research findings, we have also updated the discussion in the section. Please refer to lines 527–535, 589–593.
