# Supplementary material for: A new shortcut for competitive sports development? The purpose of and strategy for developing and introducing sepaktakraw in Taiwan
Source: PLoS One. 2025 Jan 13;20(1):e0317451. doi: 10.1371/journal.pone.0317451 (PMC11729932; doi:10.1371/journal.pone.0317451)
Supplement: Attachment — Submitted filename: Response to Reviewers.docx [file pone.0317451.s002.docx]

**Response to Reviewers**

Reviewer #2: Dear Author！
After a careful review of this article. After reading through the article, the argumentative process is too uncritical and there are areas that need to be revised.

| **Reviewer’s comments to the author** | **Response and amendments to reviewer' comments** |
| --- | --- |
| 1. The deepening of the theoretical framework is an important part of ensuring that the research is scholarly and theoretical. In further generalizing and expanding the existing theoretical framework, a more in-depth literature review of the existing research is needed to highlight the importance and innovation of this study in the current field of academic research. To further provide the theoretical and scholarly nature, a more in-depth literature review of existing studies is recommended to highlight the importance and innovations of this study in the current field of scholarly research. | Your suggestions are greatly appreciated. To provide a deeper understanding of the existing theoretical framework, we have re-examined the relevant literature and added relevant content to the literature review section. Corrections can be found on lines 114-122. |
| 2. Combined with the analysis of articles in related fields, it can refer to the relevant literature on public participation and public complaints to enhance the theoretical support of the introductory section, highlight the theoretical orientation, focus on the argument from the theoretical perspective, and present the relevant theoretical support. The following articles can be highlighted for reference: ①Sun, T., Di, K., & Shi, Q. (2024). Digital economy and carbon emission: The coupling effects of the economy in Qinghai region of China. Heliyon, 10(4). ②Sun, T., Di, K., Shi, Q., Hu, J., & Zhang, X. (2024). Study on the development path of low-carbon retail clusters empowered by digital empowerment. Journal of Retailing and Consumer Services, 81, 104006. ③Wang, J., Qiao, L., Zhu, G., Di, K., & Zhang, X. (2025). Research on the driving factors and impact mechanisms of green new quality productive forces in high-tech retail enterprises under China's Dual Carbon Goals. Journal of Retailing and Consumer Services, 82, 104092. ④Xue, H., Cai, M., Liu, B., Di, K., & Hu, J. Sustainable development through digital innovation: Unveiling the impact of big data comprehensive experimental zones on energy utilization efficiency. Sustainable Development. ⑤Liu, S., Cai, Q., Wang, M., & Di, K. (2024). Urban public services and fertility intentions of internal migrants in China. Plos one, 19(3), e0300345. ⑥Cai, Q., Chen, W., Wang, M., & Di, K. How Does Green Finance Influence Carbon Emission Intensity? A Non-Linear fsQCA-ANN Approach. Polish Journal of Environmental Studies. | Thanks for your suggestions. Following the reviewer’s recommendation, we have included citations in the introduction to articles in the relevant fields and rediscussed the research gaps. For changes, please refer to lines 50-56. |
| 3. To increase the framework diagram of this article to enhance the readability and wholeness of the level of the article. In order to enhance the readability and wholeness of the level of the article, the framework diagram of this paper can be added. The framework diagram clearly displays the existing theoretical framework, the extension and innovation points of this study, and the research methodology, which helps readers to understand the study more intuitively. The addition of such diagrams not only improves the visual effect of the article, but also makes the complex theoretical framework easier to understand. | Thank you very much for your suggestions. To improve the visual appeal of the article, we added a diagram of the framework of this article to the introduction section as suggested by the reviewers (Table 1). The nine pillars of the SPLISS model and a summary of the case results (Table 3) have been added to the literature review and findings sections. Please refer to lines 64-68, 101-102, and 318-322 for corrections. |
| 4. Strengthen the revision of academic language to improve the rigor and scientificity of academic papers. In order to improve the rigor and scientificity of academic papers, it is necessary to strengthen the revision of academic language. The academic level of the paper can be improved by refining the expression, ensuring the accuracy of terminology and logical rigor. Avoiding colloquial expressions and using appropriate academic terminology and structured sentences can help highlight the theoretical and academic nature of the research. In addition, maintaining a consistent academic style and standardized citation are key to enhancing the quality of the paper. | Thank you for the suggestion. We have amended the article to make it more academic by re-examining the terminology throughout. The article has also been reviewed by an academic editing company for accuracy, scholarship, and consistency in terminology and citations. |
